# Endoscopic Outcomes and Inflammatory Marker Correlation in Adult Patients with Corrosive Substance Ingestion

**DOI:** 10.3390/jcm14186663

**Published:** 2025-09-22

**Authors:** Seymur Aslanov, Ali Senkaya, Nalan Gulsen Unal, Cengiz Karahanlı, Idris Kurt, Ferit Celik, Alper Uysal, Ozan Fatih Sarıkaya, Ahmet Omer Ozutemiz

**Affiliations:** 1 Department of Internal Medicine, Division of Gastroenterology, Ege University Faculty of Medicine, Izmir 35040, Turkey; seymur_aslanov@yahoo.com (S.A.); dr.senkaya@gmail.com (A.S.); drnalanunal@gmail.com (N.G.U.); drferitcelik35@yahoo.com.tr (F.C.); alper_uysal@live.com (A.U.); dr.ozansarikaya@yahoo.com.tr (O.F.S.); omer.ozutemiz@superonline.com (A.O.O.); 2 Department of Internal Medicine, Division of Gastroenterology, Tekirdag City Hospital, Tekirdag 59000, Turkey; 3 Department of Internal Medicine, Division of Gastroenterology, Trakya University School of Medicine, Edirne 22030, Turkey; idrisk8607055022@gmail.com

**Keywords:** corrosive ingestion, gastrointestinal injury, endoscopy, neutrophil-to-lymphocyte ratio, C-reactive protein, Zargar classification, non-invasive markers

## Abstract

**Background/Objectives:** Corrosive substance intake remains a significant public health concern due to its potential for severe gastrointestinal (GI) injury and associated morbidity. Early risk stratification is crucial for appropriate management, yet there is a lack of reliable non-invasive predictors of injury severity. This study aimed to evaluate the clinical characteristics of adult patients with corrosive ingestion and to investigate the correlation between inflammatory markers and endoscopic injury severity. **Methods:** In this retrospective study, 83 adult patients who underwent esophagogastroduodenoscopy (EGD) following corrosive ingestion between January 2017 and January 2021 were analyzed. Endoscopic injuries were graded using the Zargar classification and categorized as mild (grades 0–2a) or severe (grades 2b–4). Demographic, clinical, endoscopic, and laboratory data, including neutrophil-to-lymphocyte ratio (NLR) and C-reactive protein (CRP) levels, were recorded. The correlation between inflammatory markers and injury severity was assessed, and receiver operating characteristic (ROC) analysis was performed to determine diagnostic accuracy. **Results:** Among the patients, 55.4% were female with a mean age of 41.5 ± 17.3 years. Most ingestions were accidental (74.7%), with bleach being the most common agent (41%). Endoscopic injury was detected in 55.4% of patients, predominantly in the stomach and esophagus. Severe injuries were associated with longer intensive care and hospital stays, increased complication rates, and more frequent organ involvement (*p* < 0.001). A weak but statistically significant correlation was found between injury severity and both NLR (r = 0.357, *p* = 0.001) and CRP (r = 0.247, *p* = 0.024). ROC analysis revealed an NLR cut-off of 2.95 (AUC = 0.804) and CRP cut-off of 2.5 (AUC = 0.706) for predicting severe injury. **Conclusions:** Early endoscopic evaluation remains essential for assessing corrosive injury severity. However, NLR and CRP may serve as useful, non-invasive indicators in predicting injury severity, potentially aiding clinical decision-making, especially in settings where endoscopy is not readily available or is contraindicated.

## 1. Introduction

Corrosive substance ingestion and its long-term effects on the gastrointestinal (GI) tract represent a significant global healthcare problem, with higher incidence rates reported in developed countries [1,2]. Corrosive injuries are associated with considerable morbidity and mortality, largely due to the challenges in initial evaluation and medical management [3]. Household cleaning products containing various chemicals are the primary source of both accidental and intentional ingestions across all age groups [4,5]. Despite their widespread availability, regulatory control over these substances remains limited [1].

Corrosive ingestion can cause GI tract damage that may be classified as either acute or chronic, depending on the clinical presentation [6,7]. The severity of tissue injury is influenced by several factors, including the type, concentration, ingested volume, and contact duration of the corrosive agent [6,8]. Prompt assessment is essential to prevent life-threatening complications such as esophagogastric perforation or stricture formation [1]. Endoscopy plays a central role in diagnosis, prognosis, and subsequent management. In the absence of suspected perforation, early endoscopic evaluation is recommended [9,10].

Although endoscopy remains the gold standard for assessing injury severity, there is growing interest in the use of non-invasive laboratory parameters for risk stratification. However, existing studies evaluating the utility of such markers are limited and yield conflicting results [11,12,13,14].

The aim of this study was to describe the clinical characteristics of patients presenting with corrosive substance ingestion and to investigate the correlation between non-invasive inflammatory markers and the severity of endoscopic injury.

## 2. Methods

This retrospective single-center study evaluated consecutive patients presenting with acute corrosive substance ingestion to the Gastroenterology Department of Ege University Medical Faculty Hospital between January 2017 and January 2021. Of 108 initially screened patients, we included 83 adults (age ≥ 18 years) who underwent diagnostic esophagogastroduodenoscopy (EGD). Exclusion criteria comprised patient refusal of endoscopic evaluation and incomplete medical records, totaling 25 excluded cases. Some asymptomatic patients presented because the ingestion was witnessed or strongly suspected, and evaluation was performed as a precaution.

### 2.1. Data Collection and Definitions

Endoscopic findings and complication data were retrieved from our prospectively maintained electronic endoscopy database. We defined the following:

- *Early complications*: Those occurring within 14 days post-ingestion.
- *Late complications*: Those developing after 14 days.
- *Total hospital stay*: Combined duration of emergency department and intensive care unit (ICU) admission.

### 2.2. Injury Classification

Corrosive injuries were classified according to the Zargar endoscopic grading system [15]. For analytical purposes, we stratified patients into the following:

- *Group A (Mild injury)*: Grades 0, 1, and 2a.
- *Group B (Severe injury)*: Grades 2b, 3a, 3b, and 4.

### 2.3. Data Extraction

We systematically collected the following variables from medical records:

1. *Demographic characteristics:* Age, sex.
2. *Exposure details:*
  ○ Corrosive substance type (classified as alkali, acidic, neutral, or other).
  ○ Intent (accidental vs. intentional).
  ○ Ingestion-to-endoscopy interval.
3. *Clinical presentation:*
  ○ Admission symptoms.
  ○ Physical examination findings.
4. *Outcome measures:*
  ○ ICU and total hospital stay duration.
  ○ Systemic complications.
  ○ Laboratory parameters (neutrophil-to-lymphocyte ratio [NLR], C-reactive protein [CRP]).
5. *Psychiatric comorbidities*: Documented diagnoses. Psychiatric comorbidity was defined as a previously established psychiatric disorder documented in the patient’s medical records or confirmed by psychiatric consultation at the time of admission.

### 2.4. Substance Classification

We categorized ingested substances as follows:

- *Alkali*: Bleach and cleaning agents containing sodium hypochlorite, ammonium hydroxide, or sodium hydroxide.
- *Acidic*: Decalcifiers and cleaners with hydrochloric acid, ammonium sulfate, hydrogen peroxide, or acetic acid.
- *Neutral*: Thinners and potassium permanganate.
- *Other*: Substances with unknown composition.

All patients provided written informed consent prior to undergoing esophagogastroduodenoscopy (EGD). Procedural preparation included pharyngeal anesthesia with 10% lidocaine spray (Xylocaine^®^ 10%, AstraZeneca, Sweden) followed by intravenous sedation using midazolam (Dormicum^®^, Roche, Switzerland) and/or propofol (Propofol 1% Fresenius^®^, Fresenius Kabi, Austria).

All endoscopic procedures were performed using a high-definition video gastroscope (Olympus GIF-HQ190, Tokyo, Japan) under one of the following conditions:

1. Performed directly by a board-certified gastroenterologist.
2. Conducted by a gastroenterology fellow under the direct supervision of an attending gastroenterologist.

All procedures were assisted by a dedicated endoscopy nurse to ensure patient safety and procedural efficiency.

Ethical approval for this study was obtained from the Ege University Faculty of Medicine Clinical Research Ethics Committee (approval no: 21-2.1T/51).

Statistical analyses were conducted using SPSS version 22 (IBM Corp., Armonk, NY, USA). Descriptive statistics for numerical variables were expressed as mean ± standard deviation (SD), median (minimum–maximum), while categorical variables were summarized as frequencies and percentages. The Kolmogorov–Smirnov test was employed to assess data normality.

For intergroup comparisons, the Kruskal–Wallis test (non-parametric numerical variables) and chi-square test (categorical variables) were applied. Post hoc pairwise comparisons were performed using the Mann–Whitney U test (numerical variables) or chi-square/Fisher’s exact test (categorical variables), with Bonferroni correction for multiple comparisons.

Spearman’s rank correlation test was used to evaluate the association between serum NLR/CRP levels and the severity of corrosive substance injury. To assess the diagnostic utility of NLR and CRP in predicting injury severity, receiver operating characteristic (ROC) curve analysis was performed, with optimal cutoff values determined by maximizing the Youden index. Corresponding sensitivity, specificity, and area under the curve (AUC) were reported.

A two-tailed alpha level of 0.05 was set as the threshold for statistical significance.

## 3. Results

Among the 83 patients included in the study, 46 (55.4%) were women, and the mean age was 41.5 ± 17.3 years (range: 17–84 years). Sixty-two patients (74.7%) had ingested the corrosive substance accidentally, with bleach being the most commonly ingested agent, reported in 34 patients (41%). The volume of ingested substance was 0–50 mL in 53 patients (63.9%), 50–100 mL in 24 patients (28.9%), and more than 100 mL in 6 patients (7.2%). Thirty-three patients (39.8%) were asymptomatic at presentation, while the most common presenting symptom was sore throat, observed in 18 patients (21.7%). Physical examination was unremarkable in 50 patients (60.2%), with oropharyngeal hyperemia being the most frequent pathologic physical examination finding (26.5%). Endoscopy was performed within the first 24 h of presentation in 77 patients (92.8%). Seventeen patients (20.5%) required hospitalization. The median duration of total hospital stay was 1 day (range: 1–28 days), and the median duration of ICU stay was 0 days (range: 0–25 days). No systemic complications or mortality were observed. Conservative treatment was administered to 98.8% of the patients. Early complications, defined as adverse events occurring within 14 days after ingestion, were observed in four patients: two cases of perforation and two cases of bleeding. Both perforations developed after the initial endoscopic assessment, and no grade-4 (perforation at the time of endoscopy) injuries were observed. Both patients with perforation required surgical intervention and subsequently recovered without mortality. Demographic and clinical characteristics are presented in Table 1.

Endoscopic examination revealed no injury in 37 patients (44.6%), while 46 patients (55.4%) had gastrointestinal tract injuries. As expected, the esophagus and the stomach were the most commonly affected sites of injury. The distribution of injuries in the esophagus, stomach, and duodenum according to the Zargar classification is shown in Table 2.

When clinical data were analyzed based on the type of ingested corrosive substance, statistically significant differences were observed between the groups in terms of total hospital stay, ICU stay, occurrence of early complications, and hospitalization rates (*p* = 0.009, 0.006, 0.002, 0.007, and 0.003, respectively). Patients who had ingested acidic substances had longer ICU and hospital stays, more severe endoscopic injuries, less frequent early complications, and higher rates of ICU admission (Table 3).

When demographic and clinical data were evaluated according to the severity of injury, statistically significant differences were found between the groups in terms of ICU stay duration, total hospital stay duration, occurrence of early and late complications, organ involvement, and ICU admissions (*p* < 0.001, <0.001, <0.001, 0.001, <0.001, and <0.001, respectively). In patients with grade 2b, 3a, 3b, and 4 injuries, both ICU and total hospital stays were significantly longer. Early and late complications were more frequent, with esophageal and gastric involvement being the most common findings (Table 4). A significant correlation was also observed between the severity of corrosive injury and the presence of symptoms and physical examination findings at presentation (*p* = 0.034 and *p* < 0.001, respectively).

**Figure 1:**
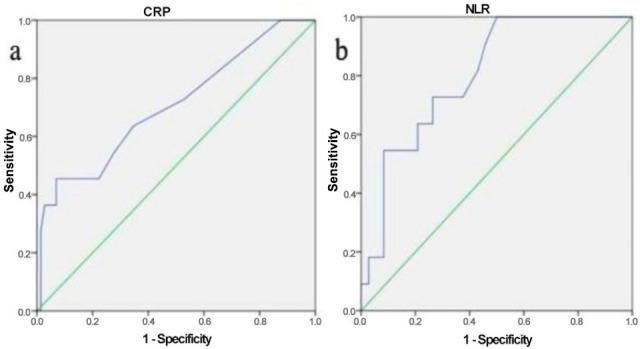
Receiver Operating Characteristic (ROC) Analysis of Biomarkers for Predicting Severe Corrosive Injury. (**a**) ROC curve demonstrating the diagnostic performance of neutrophil-to-lymphocyte ratio (NLR) in discriminating between mild (Zargar grades 0–2a) and severe (grades 2b–4) injuries. The area under the curve (AUC) was 0.804 (95% CI: 0.687–0.920), with an optimal cutoff of 2.95 (sensitivity 72.7%, specificity 73.6%). (**b**) ROC curve showing the predictive capability of C-reactive protein (CRP) for severe injuries, with an AUC of 0.706 (95% CI: 0.527–0.885). The diagonal line represents the reference line of no discrimination.

A weak but statistically significant correlation was observed between the severity of corrosive injuries and both NLR (r = 0.357, *p* = 0.001) and CRP (r = 0.247, *p* = 0.024). Receiver operating characteristic (ROC) analysis was performed to assess the predictive value of these markers for injury severity. The area under the curve (AUC) for NLR was 0.804 (95% CI: 0.687–0.920, *p* = 0.001), with a cut-off value of 2.95, yielding a sensitivity of 72.7% and a specificity of 73.6% for distinguishing between mild (grades 0, 1, 2a) and severe (grades 2b, 3a, 3b, 4) injuries (Figure 1a). For CRP, the AUC was 0.706 (95% CI: 0.527–0.885, *p* = 0.029), and the optimal cut-off value was 2.5, with a sensitivity of 63.6% and a specificity of 65.3% (Figure 1b).

## 4. Discussion

Corrosive substance ingestion is not only a life-threatening public health issue worldwide but also a significant social concern. Its true incidence remains unclear, likely due to substantial underreporting across many regions [16,17]. Corrosive ingestion is a medical emergency that often necessitates prompt endoscopic evaluation; however, the optimal timing of endoscopy remains a topic of debate. Current recommendations suggest performing endoscopy within the first 12–48 h, with the procedure considered safe up to 96 h after ingestion [17]. Nevertheless, early endoscopy remains the cornerstone of effective management [18,19]. In our study, endoscopy was performed within the first 24 h in 92.8% of patients, and 65 patients (78.3%) were discharged directly from the emergency department following the procedure. As a tertiary care center, our healthcare team maintains a high level of vigilance in the management of corrosive ingestion. This allows for the prompt identification of serious injuries and facilitates the early management of complications, while also avoiding unnecessary hospitalizations through the safe discharge of patients with no or mild injuries. We believe this approach is both clinically effective and cost-efficient.

The most commonly ingested corrosive substances are typically classified as either acids or alkali. Bathroom cleaners and dishwashing agents containing sodium hydroxide are generally alkaline, whereas toilet cleaners containing sulfuric or hydrochloric acid are typically acidic [16,20,21]. In a report of the American Association of Poison Control Centers published in 2013, sodium hypochlorite, which is a natural alkaline content in household-type bleach, was defined as the most common corrosive substance [22]. It was noted in our study that the majority of patients (74.7%) had taken the corrosive substance accidentally, and the most commonly ingested substance (41%) was bleach (sodium hypochloride). Alkaline contents were also shown to be the most common cause of corrosive injuries in similar reports from European countries [16]. As was reported in the study carried out in our country by Acehan S. et al., based on the information obtained from patients and their relatives, a great majority of our patients had ingested diluted sodium hypochloride (bleach) stored in a tea or water glass for cleaning purposes, accidentally thinking that it was drinking water [23]. Rates of accidental intake of corrosive substances were also comparable in another study carried out in our country [24].

Clinical presentations of corrosive substance intake are diverse and are not always correlated with the degree of injury [1]. In our study, the most frequent symptom observed in patients at presentation was sore throat, and the most frequent pathologic physical examination sign was oropharyngeal hyperemia. However, symptoms and physical examination may not be reliable to determine the severity and degree of the injury in the upper GI tract due to corrosive substance intake [16,25,26]. Physical examination findings were normal in 60.2% of our patients, and although 39.8% had no symptoms, 55.4% had GI tract injuries; therefore, absence of local oropharyngeal changes and being asymptomatic cannot rule out severe GI tract injury in patients who ingest corrosive substances [27]. In another study, 37% of patients without visible oropharyngeal injury were reported to have grade 2 and 3 endoscopic esophageal injury according to Zargar classification [8]. When the correlation between symptoms and physical examination findings and severity of injury was analyzed in our study, a significant correlation was found. Patients with severe injuries had more symptoms and physical examination signs (90.9% and 90.9%, *p* = 0.034 and *p* < 0.001, respectively).

Liquid corrosive substances rapidly pass from the oropharynx and usually cause more injuries in the esophagus and middle and distal parts of the stomach [1,10,28,29]. Probably as a result of this passage, isolated stomach and stomach with esophagus were affected most frequently in our patients with injuries. Longer exposure in the stomach due to the pyloric spasm caused by the ingested substance can lead to more severe injuries in the stomach [30]. In our study, duodenum injury was seen at a low rate. Pylorospasm due to the corrosive substance and neutralization of duodenal contents are considered to play a role in reducing the damage in the duodenum [31].

Determination of the injury through endoscopy is highly important for prognosis and management. Usually, Zargar grade 0 and 1 endoscopic lesions do not lead to late complications like esophageal strictures or gastric outlet obstructions. Degree of esophageal injury in endoscopy is a direct predictor of systemic complications and death, and an increase in the degree of injury is associated with a 9-fold increase in morbidity and mortality [15]. When demographic and clinical data were evaluated according to the severity of injury, duration of ICU stay and total hospital stay were longer, early and late complications were more frequent and both esophagus and stomach were affected most frequently in our patient group with ≥grade 2b severity of injury. Studies have shown that late complications are observed in patients with severe injuries, and incidences of strictures following Zargar grade 2b and grade 3 esophageal injuries can be 71% [15] and 100% [32,33], respectively. Patients with grade 2a or less injury can usually be discharged on the same day by allowing oral intake and with antacid therapy. In cases with more severe injuries (≥grade 2b), close monitoring in the intensive care unit and adequate parenteral nutrition support are required [17]. As was the case in our study, the more severe the injury, the longer it takes to stay at the hospital.

There is a consensus about the fact that caustic tissue injury is simultaneously related to the type, commercial formulation, pH, dose, concentration, viscosity, and contact time with the substance [16,17,34,35,36]. When GI tract injury is considered according to the type of corrosive substance, the conventional view is that acids preferably harm the stomach. While low surface tension and formation of protective esophageal eschar allow acids to pass without harming the esophagus to a great extent, the stomach is affected more severely. Conversely, alkalis harm the esophagus more. Higher surface tension of alkalis, which allows longer contact with the esophageal tissues and neutralization of gastric injury by acidic stomach contents, explains the more severe injury of the esophagus [1]. On the other hand, no significant correlation was found between the type of corrosive substance and upper GI tract involvement in our study (*p* = 0.310). But endoscopic injury was found to be more severe (≥grade 2b) and early complications were more frequent in patients using acidic substances, and the patients were hospitalized more frequently. Potent caustics (pH < 2 or >12) cause more rapid and severe injury [17,31,37]. It may be considered that severely injured patients in our study probably ingested potent acidic contents, but pH values of the ingested corrosive substances are not known; therefore, a clear statement cannot be made on this matter.

In our study, a weak correlation was found between NLR(r = 0.357, *p* = 0.001) and CRP(r = 0.247, *p* = 0.024) values and the severity of injury that we analyzed in order to demonstrate the correlation between non-inflammatory markers and severity of endoscopic injury. Cut-off value for NLR was 2.95 between mild and severe injury (sensitivity of 72.7% and specificity of 73.6%), and it was 2.5 for CRP (sensitivity of 63.6% and specificity of 65.3%). NLR is an important marker of systemic inflammation and is a cost-effective and easily accessible method [38]. Elevated NLR levels are associated with poor survival and increased morbidity in various chronic diseases [39]. At the time of esophageal injury due to corrosive intake, high levels of free radicals and low levels of antioxidant reserves are formed due to oxidative stress, and this results in a change in NLR [40,41,42,43,44]. In a similar study, although there was a similarly weak correlation, the correlation between NLR and CRP values and severity of injury was significant. An NLR of > 8.71 was a good diagnostic marker in distinguishing between mild and severe caustic injuries (grade 0, 1, 2 versus 3, 4) [AUC: 0.914, 95% CI (0.85–0.96, *p* < 0.001)] [45]. Our study is one of the limited number of studies evaluating NLR values and caustic injuries in the literature [45,46], and this significant positive correlation can be used as a non-invasive marker to estimate the severity of caustic injuries in the future.

The limitations of our study are its retrospective nature, lack of knowledge about the contents of some of the ingested substances, and, additionally, the inability to make a comment about potent acid or potent alkali due to lack of knowledge about the pH status.

## 5. Conclusions

In conclusion, injuries related to corrosive substance intake constitute a serious problem both for the patients and the healthcare system. Performance of endoscopy at the earliest stage possible after the corrosive substance intake has critical importance regarding the treatment and prognosis. Non-invasive markers like NLR and CRP can be used for the prediction of the severity of injury, avoiding endoscopic procedures in patients with mild injury.

## Figures and Tables

**Table 1:** Demographic and Clinical Characteristics of Patients with Corrosive Ingestion.

	Mean ± SD Median (Range)	*n* (%)
**Age (years)**	41.5 ± 17.3 40 (17–84)	
**Hospitalization Duration**		
ICU stay (days)	1.8 ± 0.6 0 (0–25)	
Total hospital stay (days)	2.9 ± 4.7 1 (1–28)	
**Laboratory Markers**		
CRP (mg/L)	5.7 ± 11 2 (0–58)	
NLR	3.5 ± 4.5 2.3 (1.1–36)	
**Gender** (female)		46 (55.4)
**Psychiatric comorbidity**		15 (18.1)
**Ingestion Characteristics Intent;**		
Accidental [f/m]		62 (74.7) [35/27]
Suicidal [f/m]		21 (25.3) [11/10]
**Corrosive substance:**		
Bleach		34 (41)
Decalcifier		10 (12.1)
Muriatic acid		9 (10.8)
Thinner		4 (4.8)
Detergent		3 (3.6)
Disinfectant		2 (2.4)
Other ^a^		21 (25.3)
**Substance type**		
Alkali		37 (44.6)
Acid		20 (24.1)
Neutral		8 (9.6)
Other		18 (21.7)
**Amount of substance ingested**		
0–50 cc		53 (63.9)
50–100 cc		24 (28.9)
Above 100 cc		6 (7.2)
**Clinical presentation**		
**Symptoms at presentation**		
No symptoms		33 (39.8)
Sore throat		18 (21.7)
Abdominal pain		4 (4.8)
Retrosternal burning		4 (4.8)
Vomiting		3 (3.6)
Chest pain		3 (3.6)
More than one symptom		14 (16.9)
Other ^b^		4 (4.8)
**Physical examination signs**		
Normal		50 (60.2)
Oropharyngeal hyperemia		22 (26.5)
Epigastric tenderness		2 (2.4)
Roughness in lung sounds		1 (1.2)
More than one sign		8 (9.7)
**Time of endoscopy**		
0–24 h		77 (92.8)
24–48 h		5 (6)
Over 48 h		1 (1.2)
**Outcome**		
Discharged from the emergency unit		65 (78.3)
Hospitalization		17 (20.5)
Discharged voluntarily		1 (1.2)
**Early complications**		
None		79 (95.2)
Perforation		2 (2.4)
Bleeding		2 (2.4)
**Late complications**		
None		40 (48.2)
Esophageal stricture		2 (2.4)
Gastric stricture		3 (3.6)
Unknown		38 (45.8)

SD: standard deviation; CRP: C-reactive protein (mg/L), NLR: neutrophil-to-lymphocyte ratio, f/m: female/male ratio; ^a^: solvent, machine oil, spirit vinegar, potassium permanganate, peroxide, sodium hydroxide, oil solvent, feeding bottle cleaning solution, FX35 car wash solution, ammonium sulphate; ^b^: coughing, respiratory distress, diarrhea, epigastric burning.

**Table 2:** Organ Involvement and Endoscopic Injury Grading in Corrosive Ingestion.

Characteristic	*n* (%)	Additional Details
**Organ involvement**		
None	37 (44.6)	
Esophagus	9 (10.8)	
Stomach	15 (18.1)	
Duodenum	0 (0)	
Esophagus + stomach	15 (18.1)	
Stomach + duodenum	1 (1.2)	
Esophagus + duodenum	1 (1.2)	
Esophagus + stomach + duodenum	5 (6)	
**Grade of esophageal injury (Zargar Grade)**		
Grade 0	52 (62.7)	Normal mucosa
Grade 1	10 (12)	Mucosal edema/hyperemia
Grade 2a	14 (16.9)	Superficial ulcers
Grade 2b	4 (4.8)	Deep focal ulcers
Grade 3a	3 (3.6)	Circumferential deep ulcers
Grade 3b-4	0 (0)	Necrosis/perforation
**Grade of gastric injury (Zargar Grade)**		
Grade 0	47 (56.6)	
Grade 1	21 (25.3)	
Grade 2a	5 (6)	
Grade 2b	2 (2.4)	
Grade 3a	7 (8.5)	
Grade 3b-4	1 (1.2)	
Grade 4	0 (0)	
**Grade of duodenal injury (Zargar Grade)**		
Grade 0	76 (91.6)	
Grade 1	2 (2.4)	
Grade 2a	3 (3.6)	
Grade 2b	2 (2.4)	
Grade 3a-4	0 (0)	

**Table 3:** Clinical Outcomes Stratified by Corrosive Substance Type.

	Alkali (*n* = 37)	Acidic (*n* = 20)	Neutral (*n* = 8)	Other (*n* = 18)	*p*-Value
**Hospitalization Duration** [Median (range)]					
Total stay (days)	1 (1–21)	1.5 (1–16)	1 (1–2)	1 (1–28)	0.009
ICU stay (days)	0 (0–20)	0 (0–16)	0 (0–25)	0 (0–2)	0.006
**Clinical Presentation [*n* (%)]**					
Asymptomatic	16 (43.2)	5 (25)	3 (37.5)	9 (50)	0.317
Symptomatic (1 symptom)	16 (43.2)	10 (50)	2 (25)	8 (44.4)	
Symptomatic (>1 symptom)	5 (13.5)	5 (25)	3 (37.5)	1 (5.6)	
**Examination sign [*n* (%)]**					
None	23 (62.2)	8 (40)	5 (62.5)	14 (77.8)	0.229
One sign	11 (26.7)	8 (40)	3 (37.5)	3 (16.7)	
More than one sign	3 (8.1)	4 (20)	0 (0)	1 (5.6)	
**Injury Severity [*n* (%)]**					
Group A (Mild)	36 (97.3)	12 (60)	7 (87.5)	17 (94.4)	0.002
Group B (Severe)	1 (2.7)	8 (40)	1 (12.5)	1 (5.6)	
**Early complication [*n* (%)]**					
No	37 (100)	16 (80)	8 (0)	18 (100)	0.007
Yes	0 (0)	4 (20)	0 (0)	0 (0)	
**Late complication [*n* (%)]**					
No	21 (56.8)	9 (22.5)	3 (37.5)	7 (38.9)	0.063
Yes	0 (0)	4 (20)	0 (0)	1 (5.6)	
Unknown	16 (43.2)	7 (35)	5 (62.5)	10 (55.6)	
**Outcomes [*n* (%)]**					
Discharged	35 (94.6)	11 (55)	13 (72.2)	7 (87.5)	0.003
Hospitalization	2 (5.4)	9 (45)	5 (27.8)	1 (12.5)	
**Treatment modality [*n* (%)]**					
Surgical	0 (0)	1 (5)	0 (0)	0 (0)	0.410
Conservative	37 (100)	19 (95)	8 (100)	18 (0)	
**Organ involvement [*n* (%)]**					
None	19 (51.4)	5 (25)	4 (50)	9 (50)	0.310
Esophagus	3 (8.1)	4 (20)	1 (12.5)	1 (5.6)	
Stomach	9 (24.3)	2 (10)	2 (25)	2 (11.1)	
Esophagus-stomach	3 (8.1)	6 (30)	1 (12.5)	5 (27.8)	
Stomach-duodenum	1 (2.7)	0 (0)	0 (0)	0 (0)	
Esophagus-duodenum	0 (0)	0 (0)	0 (0)	1 (5,6)	
Esophagus-stomach-duodenum	2 (5.4)	3 (15)	0 (0)	0 (0)	

Group A (Mild): patients with grade 0, 1, 2a injuries; Group B (Severe): patients with grade 2b, 3a, 3b, 4 injuries.

**Table 4:** Evaluation of demographic and clinical data according to the severity of injury.

	Group A	Group B	*p*-Value
**Age** [Median (range)]	39 (17–84)	41 (21–56)	0.773
**Hospitalization Duration** [Median (range)]			
Total stay (days)	1 (1–28)	8 (2–16)	<0.001
ICU stay (days)	0 (0–1)	6 (2–16)	<0.001
**Gender [*n* (%)]**			
Female	43 (59.7)	3 (27.3)	0.055
Male	29 (40.3)	8 (72.7)	
**Ingestion Characteristics** **Intent**			
Accidental	56 (77.8)	6 (54.5)	0.135
Suicidal	16 (22.2)	5 (45.5)	
**Amount of substance ingested**			
0–50 cc	48 (66.7)	5 (45.5)	0.280
50–100 cc	20 (27.8)	4 (36.4)	
>100 cc	4 (5.6)	2 (18.2)	
**Early complication**			
No	72 (100)	7 (63.6)	<0.001
Yes	0 (0)	4 (36.4)	
**Late complication**			
No	37 (51.4)	3 (27.3)	<0.001
Yes	1 (1.4)	4 (36.4)	
Unknown	34 (47.2)	4 (36.4)	
**Outcome**			
Discharge	66 (91.7)	0 (0)	<0.001
Hospitalization	6 (8.3)	11 (100)	
**Treatment modality**			
Surgical	0 (0)	1 (9.1)	0.133
Conservative	72 (100)	10 (90.9)	
**Organ involvement**			
None	37 (51.4)	0 (0)	<0.001
Esophagus	9 (12.5)	0 (0)	
Stomach	14 (19.4)	1 (9.1)	
Esophagus-stomach	9 (12.5)	6 (54.5)	
Stomach-duodenum	1 (1.4)	0 (0)	
Esophagus-duodenum	1 (1.4)	0 (0)	
Esophagus-stomach-duodenum	1 (1.4)	4 (36.4)	

Group A (mild injury): Zargar grade 0, 1, 2a; Group B (severe injury): Zargar grade 2b, 3a, 3b, 4.

## Data Availability

The original contributions presented in the study are included in the article; further inquiries can be directed to the corresponding author.

## References

[1] De Lusong M.A.A., Timbol A.B.G., Tuazon D.J.S. (2017). Management of esophageal caustic injury. World J. Gastrointest. Pharmacol. Ther..

[2] Hashmi M.U., Ali M., Ullah K., Aleem A., Khan I.H. (2018). Clinico-epidemiological Characteristics of Corrosive Ingestion: A Cross-sectional Study at a Tertiary Care Hospital of Multan, South-Punjab Pakistan. Cureus.

[3] Atiq M., Kibria R.E., Dang S., Patel D.H., Ali S.A., Beck G., Aduli F. (2009). Corrosive injury to the GI tract in adults: A practical approach. Expert Rev. Gastroenterol. Hepatol..

[4] Caganova B., Foltanova T., Puchon E., Ondriasova E., Plackova S., Fazekas T., Kuzelova M. (2017). Caustic Ingestion in the Elderly: Influence of Age on Clinical Outcome. Molecules.

[5] Mrazová K., Navrátil T., Pelclová D. (2012). Consequences of ingestions of potentially corrosive cleaning products, One-Year Follow-Up. Int. J. Electrochem. Sci..

[6] Faz A.A., Arsan F., Peyvandi H., Oroei M., Peyvandi M., Yousefi M. (2017). Epidemiologic Features and Outcomes of Caustic Ingestions; a 10-Year Cross-Sectional Study. Emergency.

[7] Haller J.A., Andrews H.G., White J.J., Tamer M.A., Cleveland W.W. (1971). Pathophysiology and management of acute corrosive burns of the esophagus: Results of treatment in 285 children. J. Pediatr. Surg..

[8] Cheng H.-T., Cheng C.-L., Lin C.-H., Tang J.-H., Chu Y.-Y., Liu N.-J., Chen P.-C. (2008). Caustic ingestion in adults: The role of endoscopic classification in predicting outcome. BMC Gastroenterol..

[9] Immaneni S., Ramamoorthy M., Rajeshwaran V.A., Murugesan M., Solomon R., Annasamy C. (2019). Clinical, Epidemiological, Endoscopic Profile And Outcome Of Corrosive Injuries Of Gastrointestinal Tract—A tertiary care experience. Int. J. Sci. Res..

[10] Radenkova-Saeva J., Loukova A., Tsekov C. (2016). Caustic injury in Adults—A study for 3 year period. Acta Medica Bulg..

[11] Kaya M., Ozdemir T., Sayan A., Arıkan A. (2010). The relationship between clinical findings and esophageal injury severity in children with corrosive agent ingestion. Ulus. Travma Acil Cerrahi Derg..

[12] Chen T.Y., Ko S.F., Chuang J.H., Kuo H.W., Tiao M.M. (2003). Predictors of esophageal stricture in children with unintentional ingestion of caustic agents. Chang Gung Med. J..

[13] Havanond C., Havanond P. (2007). Initial signs and symptoms as prognostic indicators of severe gastrointestinal tract injury due to corrosive ingestion. J. Emerg. Med..

[14] Rigo G.P., Camellini L., Azzolini F., Guazzetti S., Bedogni G., Merighi A., Bellis L., Scarcelli A., Manenti F. (2002). What is the utility of selected clinical and endoscopic parameters in predicting the risk of death after caustic ingestion?. Endoscopy.

[15] Zargar S.A., Kochhar R., Mehta S., Mehta S.K. (1991). The role of fiberoptic endoscopy in the management of corrosive ingestion and modified endoscopic classification of burns. Gastrointest. Endosc..

[16] Chirica M., Bonavina L., Kelly M.D., Sarfati E., Cattan P. (2017). Caustic ingestion. Lancet.

[17] Contini S., Scarpignato C. (2013). Caustic injury of the upper gastrointestinal tract: A comprehensive review. World J. Gastroenterol..

[18] Cabral C., Chirica M., de Chaisemartin C., Gornet J.-M., Munoz-Bongrand N., Halimi B., Cattan P., Sarfati E. (2012). Caustic injuries of the upper digestive tract: A population observational study. Surg. Endosc..

[19] Poley J.-W., Steyerberg E.W., Kuipers E.J., Dees J., Hartmans R., Tilanus H.W., Siersema P.D. (2004). Ingestion of acid and alkaline agents: Outcome and prognostic value of early upper endoscopy. Gastrointest Endosc..

[20] Kate V., Ananthakrishnan N., Kalayarasan R. (2016). Corrosive injury of esophagus and stomach. Textbook of Surgical Gastroenterology.

[21] Lakshmi C.P., Vijayahari R., Kate V., Ananthakrishnan N. (2013). A hospital-based epidemiological study of corrosive alimentary injuries with particular reference to the Indian experience. Natl. Med. J. India.

[22] Mowry J.B., Spyker D.A., Cantilena L.R., McMillan N., Ford M. (2014). 2013 Annual Report of the American Association of Poison Control Centers’ National Poison Data System (NPDS): 31st Annual Report. Clin. Toxicol..

[23] Acehan S., Satar S., Gulen M., Avci A. (2021). Evaluation of corrosive poisoning in adult patients. Am. J. Emerg. Med..

[24] Karaoğlu A., Özütemiz Ö., İlter T., Batur Y., Yönetçi N., Tekeşin O., Musoğlu A., Aydin A., Osmanoğlu N., Akarca U. (1998). Akut korozif özofajit:108 olgunun değerlendirilmesi. Turk. J. Gastroenterol..

[25] Ain Q.U., Jamil M., Abu Safian H., Akhter T.S., Batool S., Arshad M., Jamal A.M., Iqbal A., Arsh L., Abbas B. (2020). Assessing the Degree of Acute Esophageal Injury Secondary to Corrosive Intake: Insights from a Public Sector Hospitals of a Developing Country. Cureus.

[26] Chirica M., Kelly M.D., Siboni S., Aiolfi A., Riva C.G., Asti E., Ferrari D., Leppäniemi A., Broek R.P.G.T., Brichon P.Y. (2019). Esophageal emergencies: WSES guidelines. World J. Emerg. Surg. WJES.

[27] Chibishev A., Simonovska N., Shikole A. (2010). Post-corrosive injuries of upper gastrointestinal tract. Prilozi.

[28] Chibishev A., Pereska Z., Chibisheva V., Simonovska N. (2012). Corrosive poisonings in adults. Mater. Sociomed..

[29] Bremholm L., Winkel R., Born P., Suku M.L. (2009). Akut øsofageal nekrose [Acute esophageal necrosis]. Ugeskr. Laeger..

[30] French D., Sundaresan S. (2019). Caustic esophageal injury. Shackelford’s Surgery of the Alimentary Tract.

[31] Wax P.M., Yarema M., Shannon M.W., Borron S.W., Burns M.J. (2007). Corrosives. Haddad and Winchester’s Clinical Management of Poisoning and Drug Overdose.

[32] Baskın D., Urgancı N., Abbasoğlu L., Alkım C., Yalçın M., Karadağ Ç., Sever N. (2004). A standardised protocol for the acute management of corrosive ingestion in children. Pediatr Surg Int..

[33] Kay M., Wyllie R. (2009). Caustic ingestions in children. Curr. Opin. Pediatr..

[34] Hoffman R.S., Burns M.M., Gosselin S. (2020). Ingestion of Caustic Substances. N. Engl. J. Med..

[35] Hall A.H., Jacquemin D., Henny D., Mathieu L., Josset P., Meyer B. (2019). Corrosive substances ingestion: A review. Crit. Rev. Toxicol..

[36] Ramasamy K., Gumaste V.V. (2003). Corrosive ingestion in adults. J. Clin. Gastroenterol..

[37] Kirsh M.M., Ritter F. (1976). Caustic ingestion and subsequent damage to the oropharyngeal and digestive passages. Ann Thorac. Surg..

[38] Balta S., Celik T., Mikhailidis D.P., Ozturk C., Demirkol S., Aparci M., Iyisoy A. (2016). The Relation Between Atherosclerosis and the Neutrophil-Lymphocyte Ratio. Clin. Appl. Thromb Hemost..

[39] Isaac V., Wu C.Y., Huang C.T., Baune B.T., Tseng C.L., McLachlan C.S. (2016). Elevated neutrophil to lymphocyte ratio predicts mortality in medical inpatients with multiple chronic conditions. Medicine.

[40] Dundar Z.D., Ergin M., Koylu R., Ozer R., Cander B., Gunaydin Y.K. (2014). Neutrophil-lymphocyte ratio in patients with pesticide poisoning. J. Emerg. Med..

[41] Liu F., Jiang M.Z., Shu X.L., Zhang X.P. (2009). Role of oxidative stress in the pathogenesis of esophageal mucosal injury in children with reflux esophagitis. Chin. J. Contemp. Pediatr..

[42] Ranjbar A., Solhi H., Mashayekhi F.J., Susanabdi A., Rezaie A., Abdollahi M. (2005). Oxidative stress in acute human poisoning with organophosphorus insecticides; A case control study. Environ. Toxicol. Pharmacol..

[43] Kiyan G., Aktas S., Ozel K., Isbilen E., Kotiloglu E., Dagli T.E. (2004). Effects of hyperbaric oxygen therapy on caustic esophageal injury in rats. J. Pediatr. Surg..

[44] Guven A., Demirbag S., Uysal B., Topal T., Erdogan E., Korkmaz A., Ozturk H. (2008). Effect of 3-amino benzamide, a poly(adenosine diphosphate-ribose) polymerase inhibitor, in experimental caustic esophageal burn. J. Pediatr. Surg..

[45] Uyar S., Kok M. (2017). Neutrophil to lymphocyte ratio as a predictor of endoscopic damage in caustic injuries. J. Clin. Toxicol..

[46] Mathews N.V., Premkumar K., Ramamoorthy M., Chezhian A., Venkateswaran A., Shubha I. (2020). Neutrophil to lymphocyte ratio, a novel bedside predictor of endoscopic damage in corrosive gi injuries. Int. J. Sci. Res..

